# Neuroscience-related research in Ghana: a systematic evaluation of direction and capacity

**DOI:** 10.1007/s11011-015-9724-7

**Published:** 2015-09-07

**Authors:** Emmanuel Quansah, Thomas K. Karikari

**Affiliations:** Pharmacology, Faculty of Health and Life Sciences, De Montfort University, Leicester, LE1 9BH UK; Department of Molecular Biology and Biotechnology, School of Biological Science, University of Cape Coast, Cape Coast, Ghana; Neuroscience, School of Life Sciences, University of Warwick, Coventry, CV4 7AL UK; Midlands Integrative Biosciences Training Partnership, University of Warwick, Coventry, CV4 7AL UK

**Keywords:** Mental health, Neuroscience, Research capacity, Science policy, Research focus, Ghana

## Abstract

Neurological and neuropsychiatric diseases account for considerable healthcare, economic and social burdens in Ghana. In order to effectively address these burdens, appropriately-trained scientists who conduct high-impact neuroscience research will be needed. Additionally, research directions should be aligned with national research priorities. However, to provide information about current neuroscience research productivity and direction, the existing capacity and focus need to be identified. This would allow opportunities for collaborative research and training to be properly explored and developmental interventions to be better targeted. In this study, we sought to evaluate the existing capacity and direction of neuroscience-related research in Ghana. To do this, we examined publications reporting research investigations authored by scientists affiliated with Ghanaian institutions in specific areas of neuroscience over the last two decades (1995–2015). 127 articles that met our inclusion criteria were systematically evaluated in terms of research foci, annual publication trends and author affiliations. The most actively-researched areas identified include neurocognitive impairments in non-nervous system disorders, depression and suicide, epilepsy and seizures, neurological impact of substance misuse, and neurological disorders. These studies were mostly hospital and community-based surveys. About 60 % of these articles were published in the last seven years, suggesting a recent increase in research productivity. However, data on experimental and clinical research outcomes were particularly lacking. We suggest that future investigations should focus on the following specific areas where information was lacking: large-scale disease epidemiology, effectiveness of diagnostic platforms and therapeutic treatments, and the genetic, genomic and molecular bases of diseases.

## Introduction

The negative impacts of neurological and neuropsychiatric diseases on healthcare, economic and social systems have been widely reported (Dua et al. 2006; George-Carey et al. 2012; Murray and Lopez 1996; Olayinka and Mbuyi 2014; Quansah and Karikari 2015). A notable example is the 1996 Global Burden of Disease report, which showed that neuropsychiatric diseases accounted for more than a quarter of all health losses due to disability (Murray and Lopez 1996). Health loss resulting from neuropsychiatric diseases was also estimated to be twenty-fold greater than the burden of cancer, and over eight times greater than that of coronary heart disease (Murray and Lopez 1996). In Africa, dementia has been estimated to affect 2.4 % of all adults aged fifty years and above; this translated to 2.76 million people suffering from dementia as of the year 2010 (George-Carey et al. 2012). The available data also shows that an appreciable number of Africans suffer from neurological diseases, including Alzheimer’s disease, Parkinson’s disease (PD) and motor neuron diseases (Blanckenberg et al. 2013; Lekoubou et al. 2014; Quansah and Karikari 2015). These diseases represent high burdens on the continent’s economic, social and healthcare systems (Blanckenberg et al. 2013; Cilia et al. 2011; George-Carey et al. 2012). As the prevalence of neurological and neuropsychiatric diseases in Africa has been predicted to increase in the near future, the amount of disease-associated burdens are also expected to rise (George-Carey et al. 2012). Furthermore, many common diseases in Africa do have associated neurocognitive impairments; examples include malaria, tuberculosis, Human Immunodeficiency Virus/ Acquired Immune Deficiency Syndrome (HIV/AIDS) and the neglected tropical diseases (Alkali et al. 2013; Karuppiah et al. 2009; Lekoubou et al. 2014; Mireku et al. 2015; Pepper et al. 2009). Taken together, neurological and neuropsychiatric diseases, as well as neurocognitive problems associated with non-nervous system disorders, do represent major public health challenges in Africa. For this reason, improved funding and policy support from the continent’s political, economic, healthcare and scientific community towards the better understanding of disease epidemiology, aetiology and propagation is needed in order to help accelerate the development of measures aimed at disease control and treatment (Abdulmalik et al. 2014; Awenva et al. 2010; Lekoubou et al. 2014). Improved investment in neuroscience research in Africa would not only benefit the continent in terms of providing further molecular, genetic and clinical insights into neurological and neuropsychiatric diseases, but would also help to better address neurocognitive impairments associated with common non-nervous system disorders (Karikari et al. 2015a; Karikari and Aleksic 2015).

Recent studies have shown that the genetic basis of some neurological diseases (including PD, spinal muscular atrophy and amyotrophic lateral sclerosis) differ between African and non-African populations (Blanckenberg et al. 2013; Karikari and Aleksic 2015; Quansah and Karikari 2015). However, knowledge gaps exist regarding how these diseases progress among African populations, and also concerning the molecular basis of disease susceptibility and resistance among specific populations and individuals (Karikari and Aleksic 2015; Quansah and Karikari 2015). In order to address these knowledge gaps, more innovative approaches must be developed. One of the fundamental approaches might be to identify potentially more effective methods of assessing disease epidemiology (Mutabaruka et al. 2011). This would inform policymakers, health planners, scientists and the general public about the significance of the problem, potentially leading to the development of more capacity-building and cross-sector collaborative efforts to address the issues (Mutabaruka et al. 2011).

Currently, there is uneven representation of African countries and specific populations in neurological and neuropsychiatric disease epidemiology reports (Baxter et al. 2013; Blanckenberg et al. 2013; George-Carey et al. 2012; Lekoubou et al. 2014; Olayinka and Mbuyi 2014; Quansah and Karikari 2015). Global disease surveillance systems such as the United Nations Children’s Fund (UNICEF)’s Multiple Indicator Cluster Surveys, the United States Agency for International Development (USAID)-funded Demographic and Health Surveys project and the World Health Organization (WHO)’s Stepwise Approach to Chronic Disease Factor Surveillance have been using dynamic approaches to quantify the burden of non-communicable diseases in low- and middle-income countries (Bonita 2009; Corsi et al. 2012; http://mics.unicef.org). Nonetheless, epidemiology of the wide spectrum of neurological and neuropsychiatric diseases in Africa have not been fully reported in these surveys (Lekoubou et al. 2014; Quansah and Karikari 2015). This lack of epidemiological data may contribute to the poor patient support systems and the ineffectiveness of mental health- and dementia-related policies available in countries (Abdulmalik et al. 2014; Olayinka and Mbuyi 2014; Sipsma et al. 2013). One could argue that since the burdens of these diseases have not been adequately quantified, health planners and policy makers lack the scientific evidence to depend upon to make adequate resource provisions for biomedical research and healthcare (Baxter et al. 2013). This is exemplified by the evidence that many patients suffering from these diseases in several countries receive no treatment. The WHO’s World Mental Health survey, for instance, estimated that only 20 % of patients diagnosed with serious mental health disorders in Nigeria received treatment in a particular year (Wang et al. 2007). Similar healthcare gaps have also been reported from other sub-Saharan African (SSA) countries, including Ghana (Abdulmalik et al. 2014; Ferri et al. 2004). Most SSA countries have low policy priorities for neurological and neuropsychiatric diseases; research and healthcare in this area are often hampered by inadequate human resources, poor funding, as well as widespread public misconceptions regarding neurological and mental health issues (Gureje et al. 2007; Saraceno et al. 2007). There is usually a tendency to prioritise programmes targeting infectious diseases, malaria and reproductive health, leaving the neurosciences and related areas with minimal resources (Prince et al. 2007). As a result, while most of the global burden of neurological and neuropsychiatric diseases occurs in low- and middle-income countries, these countries have the least resources to address these disorders and their associated problems (Baxter et al. 2013; World Health Organization 2010).

Although Ghana is actively involved in biomedical research, neuroscience seems neglected in terms of both healthcare and research (Doku et al. 2012; World Health Organization 2010; Sipsma et al. 2013). Data on disease epidemiology, treatment options and outcomes, and biomedical research findings are lacking (Read and Doku 2012). In the absence of reliable data, attempts have been made to fill the gap with information extrapolated from international estimates; however, this approach may not provide a true representation of the situation (Read and Doku 2012). In order to develop neuroscience-related research, clinical and social care in Ghana, a few interventions have been introduced recently. These include the enactment of a mental health law by the Parliament of the Republic of Ghana (Act 846 of 2012), aimed at introducing a paradigm shift in the delivery of mental health services in the country, moving from an institution-based approach to a community-based model (Roberts et al. 2014). The law was also designed to address the disturbing social challenges of stigmatisation and discrimination against mentally-ill individuals (Roberts et al. 2014; World Health Organization 2011). Although the new law and training initiatives such as The Kintampo Project (http://www.thekintampoproject.org) provide new directions in mental health development and workforce training, additional efforts are required to provide more comprehensive in-country data and resources to address neuroscience-related issues.

The success of a country’s local research efforts depends largely on the capacity of the available research workforce (Kennard 2000; Saraceno et al. 2007). Therefore, for the existing and future efforts aimed at addressing neurological diseases and related problems in Ghana to be successful, the preparedness of resident scientists to help execute these assignments needs to be addressed. Here, we aimed to provide evidence on the capacity of scientists for neuroscience-related research in Ghana, through a systematic evaluation of the published literature over the past two decades. By using scientific publications as a measure of research productivity, we were able to analyse publication trends, research foci, as well as institutional distribution of scientists working in this area. The information provided here will serve as a resource to inform the development of neuroscience education and research in Ghana, by targeting capacity-building efforts and other support programmes to areas where the need exists.

## Methods

### Data sources and search strategy

**Fig. 1:**
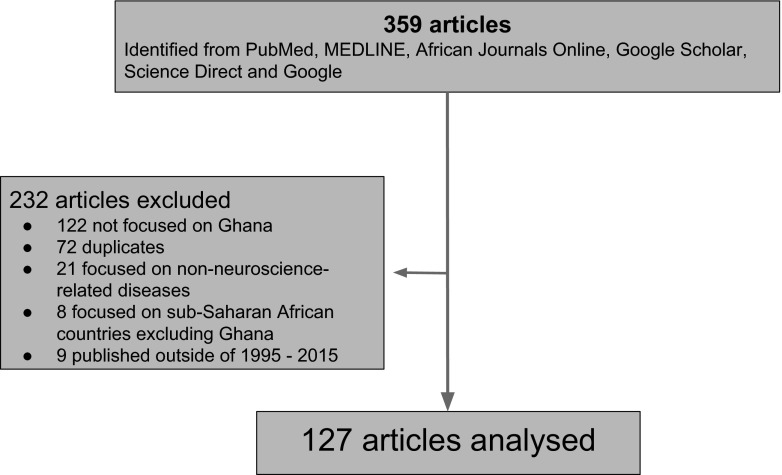
Flow diagram showing the article selection process

We searched PubMed, MEDLINE via EBSCO, African Journals Online, ScienceDirect, Google and Google Scholar for neuroscience-related studies conducted on Ghanaian subjects and those authored by scientists affiliated with Ghanaian institutions over the last two decades (1st January 1995 to 10th April 2015). We used the following search terms in combination with “Ghana”: “neurological disorders,” “mental health,” “psychiatric disorders,” “dementia,” “Alzheimer’s disease,” “Parkinson’s disease,” “autism,” “attention deficit hyperactivity disorder,” “Bipolar Disorder,” “Obsessive Compulsive Disorder,” “dyskinesia,” “depression,” “epilepsy,” “convulsion,” “seizure,” “psychological health,” “cerebrum,” “cerebellum,” “cortex” and “neuroscience”. Subsequently, we scanned the references from the articles obtained from the initial search for potential articles we might have missed. We subsequently evaluated the articles obtained (titles, abstracts, and then full texts) and screened them for inclusion (see below for inclusion criteria and Fig. 1 for article selection flow chart).

### Study selection

We selected studies conducted in Ghana that reported on any aspect of neuroscience; including neurological diseases, mental health and experimental neuroscience. No restrictions were made with regards to study design but studies not focusing on Ghana or not including Ghanaian subjects were removed. Duplicate entries were also removed.

## Results and discussion

**Fig. 2:**
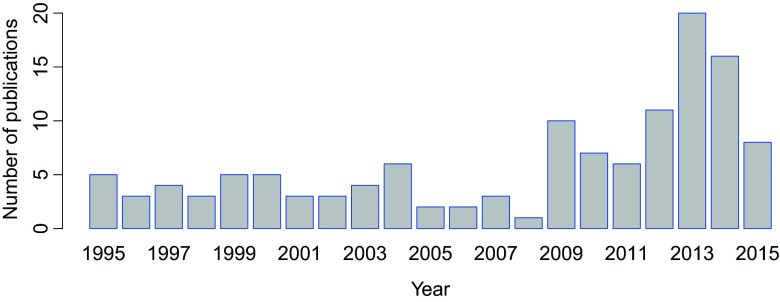
Annual distribution of neuroscience-related publications authored by scientists affiliated with Ghanaian institutions over the last two decades (1995–2015)

A total of 359 articles were identified through the literature search. These articles were evaluated and 127 publications that met the inclusion criteria were selected for further analyses (Fig. 1). Initially, the publications were grouped into themes based on their research foci. The following are the number of studies per research focus: epilepsy and seizures (9 studies), depression and suicide (14 studies), neurological disorders (6 studies), neurocognitive impairments in non-nervous system disorders (20 studies), nervous system effects of substance misuse (7 articles), and mental health among women (18 articles). Others included: the national mental health system (16 studies), support and help-seeking for mental health patients (6 studies), psychopharmacology and pharmacogenetics (3 studies), community studies such as those that investigated the relation between poverty and mental health (11 articles), as well as clinical surveys and case reports (13 articles). The percentage distribution of the number of articles for each research focus is provided in Table 1. These articles were further evaluated based on the (i) specific focus of the research reported (ii) number of articles reported under a particular research area, expressed as a percentage of the total number of articles evaluated, and (iii) annual publication trends and the institutional affliliations of Ghanaian authors (Table 1 and Fig. 2). We identified that about 60 % of the articles evaluated were published within the last seven years (Fig. 2). In the rest of this article, we will discuss in detail the epidemiology of neurological and neuropsychiatric disorders as reported from Ghana, the neuroscience-related research directions in the country, as well as the established legal and healthcare frameworks regulating neuropsychiatric service delivery. We will also discuss the existing neuroscience research capacity in the country, areas where improvement is needed and what might be required to deliver this improvement.

**Table 1:** Overview of neuroscience-related studies conducted by scientists affiliated with Ghanaian institutions over the last two decades (1995–2015)

Research area	Total number of articles (percentage)	Specific research focus	References	Ghanaian authors’ affiliations*
Epilepsy and seizures	9 (7.1 %)	Clinical features, causes and consequences of convulsive epilepsy; association of multiple parasitic infections with epilepsy; risk factors for active convulsive epilepsy; genetic risk of acute seizures.	Commey 1995 West Afr J Med 14(4):189–93; Nyame and Biritwum 1997 West Afr J Med 16(3):139–145; Owusu-Ofori et al. 2004 Int J Infect Dis 8(6):353–361; Adjei et al. 2013 Epilepsy Behav 29(2):316–321; Kariuki et al. 2013 Epilepsia 54(6): 990–1001; Kariuki et al. 2014 Epilepsia 55(1):76–85; Ngugi et al. 2013 Lancet Neurol 12(3): 253–263; Dugbartey and Barimah 2013 Ethn Dis 23(1):1–5; Kamuyu et al. 2014 PLoS Negl Trop Dis 8(5): e2908	KBTH, UG-MS, KATH, KHRC
Depression and suicide	14 (11.0 %)	Prevalence and determinants of depressive symptoms; correlation between emotional fluctuation and depression; mental health in hypertension; socio-demographic factors associated with major depressive episodes; attitudes towards suicide and its prevention among clinicians; attempted suicide - motivation, stigma and coping.	Gold et al. 2013 Int J Gynaecol Obstet 120(3):228–231; Turkson and Dua 1996 West Afr J Med 15(2):85–90; Osei 2001 Ghana Med J 35(3):111–15; Dorahy et al. 2000 J Soc Behav Pers 15(4):569–80; Okronipa et al. 2012 AIDS Behav 16(8): 2216–2225; Ambugo 2013 Soc Sci Med 113:154–160; Kretchy et al. 2014 Int J Ment Health Syst 8:25; Asante and Andoh-Arthur 2015 J Affect Disorders 171:161–166; Chan et al. 2015 J Epidemiol Glob Health 5(1):65–74; Osafo et al. 2012 Int J Nurs Stud 49:691–700; Osafo et al. 2015 Death Stud 39(5): 274–280; Hjemeland et al. 2008 Crisis 29(1):20–31; Eshun 2000 Cross-Cult Res 34(3):250–63; Eshun 2003 Suicide Life Threat Behav 33(2):165–171.	KATH, UG-MS, RUCST, UG
Neurological disorders (Parkinson’s disease, schizophrenia and dementia)	6 (4.7 %)	Motor complications in Parkinson’s disease; dietary habits of Parkinson’s disease patients; prevalence and genetics of Parkinson’s disease; worldwide societal cost of dementia; schizophrenia case study in a 23 year old; screening for *LRRK2* gene mutations in patients with Parkinson’s disease.	Barichella et al. 2013 Nutrition 29:470–473; Blanckenberg et al. 2013 J Neurol Sci 335:22–25; Cilia et al. 2014 Brain 137:2731–2742; Turkson 2000 East Afr Med J 77(11):629–630; Wimo et al. 2010 Alzheimers Dement 6:98–103; Cilia et al. 2012 J Neurol 259:569–570.	KATH, KBTH, UG-MS
Neurological problems associated with non-nervous system diseases (e.g., malaria, cancer and HIV/AIDS)	20 (15.7 %)	Delayed neuropsychiatric effects of malaria; psychological distress in Ghana; development of stroke care; psychosocial aspects of breast cancer treatment; post cerebral malaria and associated syndromes; chronic conditions and sleep problems among adults; socioeconomic burden of chronic diseases among adults; bullying and psychological health; CNS lesions caused by cerebral toxoplasmosis in HIV; spontaneous intracerebral haemorrhage.	Steele and Baffoe-Bonnie 1995 Pediatr Infect Dis J 14(4):281–285; Amedofu et al. 1997 Afr J Health Sci 4(1):29–32; Dugbartey et al. Nerv Ment Dis 186(3):183–186; Hogson et al. 2001 Int J Epidem 30:1440–1446; Obajimi et al. 2002a West Afr J Med 21(1):60–62; Obajimi et al. 2002b West Afr J Med 21(2):121–123; Armah et al. 2005 Int J Environ Res Public Health 2(1):123–131; Bedu-Addo 2006 West Afr J Med 25(3): 252–253; Akpalu and Nyame 2009 Ghana Med J 43(4):157–163; Clegg-Lamptey et al. 2009 East Afr Med J 86(7):348–353; Karuppiah et al. 2009 J Child Neurol 24(4):487–490; Dinglas et al. 2011 West Afr J Med 30(2):84–88; Gould et al. 2011 Int J Stroke 6(2):150–151; Owusu et al. 2011 J Sch Health 81:231–238; Asante 2012 Afr J Psychiatry 15:340–345; Canavan et al. 2013 Int J Ment Health Syst 7:9; Donkor et al. 2014 Clin Interv Aging 9:1701–1708; Koyanagi et al. 2014 BMJ Open 5(4):e007313; Minicuci et al. 2014 Glob Health Action 7: 21292; Essuman et al. 2010 Malaria J 9:232.	KNUST, KBTH, KATH, UG-MS, UG, Ridge Hospital, Tamale Central Hospital
Clinical surveys and case reports	13 (10.2 %)	Enteroviruses and neurological impairments; impact of blood glucose and cholesterol levels on the manifestation of psychiatric disorders; common psychiatric disorders among adults; psychiatric disorders among adolescents; misdiagnosis of alternating hemiplegia as intractable epilepsy; classical Rett syndrome.	Turkson and Asante 1997 West Afr J Med 16(2):88–92; Turkson 1996 West Afr J Med 15(1):31–35; Turkson and Asamoah 1997 West Afr J Med 16(3):146–149; Turkson 1998a East Afr Med J 75(6):336–338; Turkson 1998b East Afr Med J 75(9):556–557; Andrews et al. 2003 West Afr J Med 22(2):167–172; Owiredu et al. 2009 Pakistan J Biol Sci 12(3):252–257; Howe et al. 2013 World Neurosurg 80(6):e171-e174; Tettey et al. 2014 Pan Afr Med J 18:232; Appiah-Poku et al. 2004 Soc Psychiatry Psychiatric Epidemiol 39(3):208–211; Badoe 2009 West Afr J Med 28(2):134–136; Badoe 2011 West Afr J Med 30(2):140–144; Osei 2004 Ghana Med J 37(2):62–67.	UG, Nah-Bita Hospital, KNUST, Noguchi Memorial Institute for Medical Research, UG-MS
Community studies	11 (8.7 %)	How community physical and social stressors relate to mental health; association between sleep quality and cognitive performance; hard times and common mental disorders; population views on mental illness; occupation, poverty and mental health improvement; poor mental health in Ghana and the risk; homelessness and mental health in Ghana.	Ofori Attah and Linden 1995 Soc Sci Med 40(9): 1231–1242; Biritwum et al. 2000 Ann Trop Med Parasitol 94(8):771–8; Barke et al. 2011 Soc Psychiatr Epidemiol 46:1191–1202; de-Graft Aikins et al. 2012 Ghana Med J 46(2):59–68; Dzator 2013 J Behav Health Serv Res 71–87; Gildner et al. 2014 J Clin Sleep Med 10(6):613–621; Greif and Dodoo 2015 Health Place 33:57–66; de-Graft Aikins and Ofori-Atta 2007 J Health Psychol 12(5):761–78; Boyce et al. 2009 ALTER – Eur J Disab Res 3:233–244; Sipsma et al. 2013 BMC Public Health 13:288; Osei 2003 Ghana Med J 37(2):62–67.	UG, University of Health and Allied Sciences, BasicNeeds (Tamale), KBTH
Substance misuse	7 (5.5 %)	Drug abuse and its mental health consequences; tobacco use in adults – health risks and wellbeing; alcohol misuse among psychiatric outpatients.	Redvers et al. 2006 Prim Care Community Psychiatr 11:179–83; Lamptey 2005 Ghana Med J 39(1):2–7; Akyeampong 1995 Cult Med Psychiatr 19(2):261–80; Affinnih 1999a J Psychoactive Drugs 31(4):395–403; Affinih 1999b Subst Use Misuse (2):157–69; Turkson et al. 1996 West Afr J Med 15(1):31–35; Yawson et al. 2013 BMC Public Health 13:979.	KBTH, UG-MS
Mental health among women	18 (14.2 %)	Adverse effects of antenatal depression on mother and newborns; antepartum depression in women; symptoms of common mental disorders among women in Accra; anxiety disorder in antepartum women; depression among infertile women; psychosocial health of infertile women.	Bindt et al. 2012 PLoS ONE 7(10):e48396; Bindt et al. 2013 PLoS ONE 8(11):e80711; Thapa et al. 2014 J Psychiatry 17(6):1000167; Weobong et al. 2009 J Affect Disorders 113(1–2):109–117; Weobong et al. 2014a J Affect Disorders 165:1–7; Weobong et al. 2014b PLoS ONE 9(12):e116333; Weobong et al. 2015 Depress Anxiety 32(2):108–119; Bennett et al. 2004 Br J Psychiatry 185:312–7; Avotri and Walters 1999 Soc Sci Med 48:1123–33; Avotri and Walters 2001 J Gend Stud 10(2):197–211; Ofori-Attah et al. 2010a Int Rev Psychiatry 22(6):589–59; de Menil et al. 2012 Ghana Med J 46(2):95–103; Gardner et al. 2013 Midwifery 30(6):756–763; Guo et al. 2013 Am J Epidemiol 178(9):1394–1402; Guo et al. 2014 BMC Psychiatry 14:156; Naab et al. 2013 J Nurs Scholarship 45(2):132–140; Alhassan et al. 2014 BMC Womens Health 14:42; Barthel et al. 2014 J Affect Disorders 169:203–211.	KNUST, KATH, KHRC, UG-MS, UG
Mental health system	16 (12.6 %)	Mental health policy development and implementation; mental health services and legislation; overview of Ghana’s mental health system; implementing the mental health act in Ghana and the challenges ahead; mental health leadership and advocacy; historical survey of psychiatric practice in Ghana; review of mental health research.	Laugharne and Burns 1999 Psychiatr Bull 23(6):361–63; Laugharne et al. 2009 Acad Psychiatry 33(1):71–5; Ferri et al. 2004 Soc Psychiatry Psychiatr Epidemiol 39(3):218–227; Flisher et al. 2007 J Health Psychol 12(3):505–16; Ofori-Attah et al. 2010 Afr J Psychiatry 13(2):99–108; Kleintjes et al. 2010 Afr J Psychiatry 13:132–139; Fournier 2011 Berkeley Undergrad J 24(3); Roberts et al. 2014 Int J Ment Health Syst 8:16; Lund et al. 2015 Epidemiol Psychiatr Sci 24(3):233–240; Adjorlolo 2015 Appl Neuropsychol Adult 26:1–11; Abdulmalik et al. 2014 Int J Ment Health Syst 8(1):5; Doku et al. 2012 Ghana Med J 46(4):241–251; Skeen et al. 2010 Int Rev Psychiatry 22(6):624–631; Asare 2012 Ghana Med J 46(3):114–115; Read and Doku 2012 Ghana Med J 46(2):29–38; Akpalu et al. 2010 Afr J Psychiatry 13(2):109–15.	UG-MS, KHRC, University of Development Studies,
Support system and help-seeking among patients	6 (4.7 %)	Sitting with others, mental self-help groups; transitioning pre-schoolers with autism to kindergarten; orientation towards help-seeking for mental disorders.	Fosu 1995 Soc Sci Med 40(8):1029–1040; Appiah-Poku et al. 2004 Soc Psychiatry Psychiatr Epidemiol 39:208–11; Read et al. 2009 Glob Health 5(1):13; Quinn 2007 Int J Soc Psychiatry 53(2):175–88; Denkyirah and Agbeke 2010 Early Child Educ J 38:265–270; Cohen et al. 2012 Int J Ment Health Syst 6:1.	KNUST, University of Education
Psychopharmacology and pharmacogenetics	3 (2.4 %)	Pharmacogenetics of catechol-O-methyltransferase in Ghanaians; psychotropic drug prescriptions in Accra; drug compliance among psychiatric patients.	Sanati 2009 Int Psychiatry 6(3):69–70; Mensah and Yeboah 2003 Ghana Med J 37(2):68–71; Ameyaw et al. 2000 Hum Mutat 16(5):445–456.	UG, KATH
Herbal medicine	4 (3.1 %)	Anti-convulsant effects of *Synedrella nodiflora* and *Antiaris toxicara*; antidepressant effects of *Kalanchoe integra* leaf extract.	Amoateng et al. 2012 J Pharm Bioallied Sci 4(2):140–148; Gyamfi et al. 1999 Hum Exp Toxicol 17(8):418–423; Mante et al. 2013 ISRN Pharmacol 519208; Kukuia et al. 2015 J Pharm Bioallied Sci 7(1):26–31.	KNUST, UG, University of Cape Coast
Total number of articles	127			

*CNS central nervous system, *HIV* human immunodeficiency virus, *RUCST* Regent University College of Science and Technology, *UG* University of Ghana, *UG-MS* University of Ghana Medical School, *KHRC* kintampo health research center, *KATH* Komfo Anokye Teaching Hospital, *KBTH* Korle Bu Teaching Hospital, *KNUST* Kwame Nkrumah University of Science and Technology

### Epidemiology of neurological and neuropsychiatric diseases

Initial studies, conducted over five decades ago, predicted future increases in the burden of psychiatric disorders in Ghana due to anticipated impacts of industrialisation and acculturation (Field 1958). However, it remains largely unknown whether these predictions have been achieved or not. This is because recent epidemiological studies have focused mainly on small sections of the population and have relied almost exclusively on clinical surveys (Read and Doku 2012). While the review of hospital records provides important information about disease epidemiology, hospital cases alone may not be reliable indicators of disease prevalence, especially in African settings where (i) many people have difficulties accessing healthcare (ii) the under-resourced and over-stretched nature of healthcare facilities means that not all patients can be adequately attended to (iii) record keeping in health facilities is sometimes poor, and (iv) appreciable portions of the population depend on herbal and traditional medicine for their healthcare needs (Ferri et al. 2004; Nguta et al. 2015; Roberts et al. 2014; van Andel et al. 2012). Larger-scale and more diverse epidemiological studies combining data from multiple sources are therefore required. This would help to inform new decisions in health policy, preventive healthcare, evidence-based medical practice as well as better-targeted biomedical research aimed at identifying disease risk factors and potential therapeutic targets and agents (Quansah and Karikari 2015).

One of the major studies identified in terms of disease epidemiology was a retrospective study conducted into the prevalence of psychiatric disorders among adolescents attending a psychiatric out-patient clinic in Accra from 1987 to 1994 (Turkson 1996). Out of the 454 adolescents (239 females) whose records were reviewed, 269 patients (59.3 %) were reportedly diagnosed with psychiatric illnesses. Out of these 269 patients, 88 were diagnosed with functional psychoses (including depression and psychoneurotic disorders), 55 were diagnosed with personality disorders, 27 were diagnosed with organic psychoses and 36 were suffering from other kinds of psychiatric disorders (Turkson 1996).

A frequently-used means to assess disease occurrence and people’s perceptions about diseases is self-reported community surveys (Hunt and Bhopal 2004). Community-based studies reporting on the prevalence of psychiatric disorders and related diseases have been conducted in Ghana, although the use of standardised epidemiological methods have been lacking in some of these investigations (Ferri et al. 2004; Field 1958). By using a self-reported questionnaire and mental state examinations, Osei (2003) interviewed 194 study participants in Kumasi, the capital of the Ashanti region. It was identified that five women who participated in the study were suffering from schizophrenia, five men were suffering from somatisation and 38 others (including 33 women) were living with depressive illness. A psychiatric illness prevalence rate of 27.51 % was reported from this study. More of such community-based surveys conducted on larger scales are urgently needed to help in the estimation of disease prevalence. This is particularly necessary due to the fact that many Ghanaians seek healing from neurological and psychiatric conditions from spiritual centres, meaning that such people would be missed in clinical record surveys (Turkson 2000).

### The focus of research on neurological and neuropsychiatric diseases in Ghana

Research on neurological diseases in Ghana is limited. The available studies in this area reported on PD (three studies), economic impacts of dementia and a clinical report on a schizophrenic patient (one study each). Only one study focused on the genetic aspect of a disease. In that study, 54 patients suffering from PD and 46 matched controls were screened for the presence of the G2019S pathogenic mutation in the leucine-rich repeat kinase 2 (*LRRK2*) gene, which has been associated with familial PD (Cilia et al. 2012). However, this mutation was not found in the genome of both the patients and controls, suggesting that the genetic basis of familial PD among Ghanaians might differ from people elsewhere (Cilia et al. 2012). This finding corroborated earlier reports from Nigeria and South Africa that showed that the *LRRK2-G2019S* pathogenic mutation was missing among study subjects (Bardien et al. 2010; Okubadejo et al. 2008). These results support the growing body of evidence suggesting that the genetic basis of specific neurological diseases is population-specific, justifying the need for further studies into how these diseases progress among African populations (Karikari and Aleksic 2015; Quansah and Karikari 2015). Given that modern humans were believed to have originated from Africa, increasing the involvement of African populations in disease-focused genetic and genomic studies might provide novel information that would shape the future of neurological research and healthcare on the continent (Karikari and Aleksic 2015).

Another disease that has received research attention in Ghana is epilepsy. In 1995, it was reported that convulsive disorder accounted for 3 % of outpatient visits to the pediatric department of Ghana’s biggest tertiary hospital, Korle Bu teaching hospital, over a decade (Commey 1995). 51.5 % of these patients were enrolled in the hospital’s pediatric neurodevelopmental clinic (Commey 1995). In examining data on active convulsive epilepsy from five African countries including Ghana, Kariuki et al. (2014) identified some important features (seizure types, neurologic deficits, encephalopathy) and co-morbidities (malnutrition, cognitive impairment). Based on the findings, it was proposed that these features should be integrated into the management of epilepsy. Even though epilepsy is one of the most-studied neuropsychiatric disorders in Ghana, the available investigations were mostly clinical reports; non-hospital-based estimations of disease prevalence, potential causes and public perceptions about the disease were lacking. One of the largest community surveys on epilepsy was conducted by Nyame and Biritwum (1997). Upon interviewing 380 participants in Accra, the researchers reported that while almost all the people sampled could accurately describe an epileptic person, 172 (45.3 %) of them did not know the cause of epilepsy and 37.6 % did not know how it could be treated (Nyame and Biritwum 1997). It was further reported that out of the 358 responses about the causes of epilepsy, 114 (31.8 %) respondents believed that epilepsy was an inherited disease while 100 (27.9 %) believed that it had spiritual causes.

Furthermore, efforts have been directed at estimating the prevalence of depression among Ghanaians, particularly among women. In a cross-sectional analysis using data from a national representative survey conducted in 2009–2010 (involving  9,524 participants), the Kessler Psychological Distress scale was used to measure psychological distress (Sipsma et al. 2013). An overall psychological distress rate of 18.7 % was reported among the participants, with 11.7 and 7.0 % reporting either moderate or severe psychological distress respectively. Moreover, it has recently been reported that out of 270 university students (138 females) interviewed using the Center for Epidemiological Studies Short Depression Scale (CES-D10), overall prevalence of depression among university students in Ghana was estimated to be 39.2 %, with 31.1 and 8.1 % having either mild or severe depressive symptoms respectively (Oppong Asante and Andoh-Arthur 2015). Given the cross-sectional nature of most of these studies, increased numbers of participants may be required in order to make future findings more representative.

Substance misuse is another area that has been given research attention. Studies here have been focused principally on the neuropsychiatric effects of tobacco and alcohol use. With regard to this, Yawson et al. (2013) estimated the prevalence of daily tobacco smoking among adults in Ghana to be 7.6 %. This data was generated from 4305 study subjects aged 50 years and above.

Other publications were focused on mental health among women. These studies reported specifically on antenatal depression, antepartum depression, anxiety disorders and depression among infertile women (Table 1). In addition, a few other publications reported on the following: the relation between poverty and mental health, psychopharmacology, the use of herbal medicine in treating neuropsychiatric conditions, and help-seeking and support system for patients (Table 1).

Overall, it is worth noting that no research work has been conducted on diseases such as Alzheimer’s disease, fronto-temporal dementia, bipolar disorders, Huntington’s disease, dyskinesia as well as neurodevelopmental disorders such as autism and attention deficit hyperactivity disorder. Hence, the prevalence, causes (both genetic and sporadic) and patient care platforms for these disorders in Ghana have not been evaluated. Moreover, reports on the genetic, genomic and molecular underpinnings of common neurological diseases and neurological aspects of other diseases were lacking. A plausible explanation would be that the scientific capacity for experimental neuroscience and neurology research in Ghana is low (Karikari and Quansah 2015). This viewpoint is supported by findings of the Thomson Reuter’s Global Research Report in 2010. While Ghana was the sixth best-ranked country in central Africa in terms of annual number of publications (in this report, African countries were broadly categorised into north, south and central Africa), the country was not part of the top five African countries in the neuroscience and behaviour field (Adams et al. 2010).

Appropriate mechanisms should therefore be developed to improve neuroscience research (particularly bench-science, clinical and computational aspects) in the country (Karikari and Quansah 2015).

### Publication trends and institutions conducting neuroscience-related research in Ghana

The average number of publications on neuroscience-related research from Ghana between 1995 and 2008 was about six per year. However, a change in this trend was later observed as over 60 % of the articles were published in the last seven years (2009–2015), with about 20 articles published in 2013 alone (Fig. 2). This represents a recent improvement in neuroscience-related research output in Ghana. Most of the studies were either clinical case reports or community-based studies, with only a few (4.7 %; Table 1) examining neurological and neurodegenerative disorders. In addition, about 15.7 % (Table 1) of the studies investigated the neurocognitive impairments associated with non-nervous system diseases, giving an indication that scientists in Ghana have been applying neuroscience concepts to address pressing health concerns in diseases such as HIV/AIDS, breast cancer and malaria. With most of the publications being hospital-based reviews, majority of the authors were affiliated with the two leading hospitals in Ghana, that is, the Korle-Bu teaching hospital (KBTH) and the Komfo Anokye teaching hospital (KATH; Table 1). Many other authors were affliliated with the University of Ghana Medical School, which is associated with KBTH. The remaining authors were affiliated with universities such as the Kwame Nkrumah University of Science and Technology, the University of Development Studies, the University of Cape Coast and the University of Education (Table 1). The Kintampo Health Research Center and Noguchi Memorial Institute for Medical Research were also identified as the major research centers contributing to neuroscience-related research in Ghana (Table 1).

### Ghana’s mental health system and the mental health law

There are currently three psychiatric hospitals in Ghana; these are the Accra, Pantang and Ankaful psychiatric hospitals (Roberts et al. 2014). These hospitals have a total of about 1322 beds, although only one has a children’s ward consisting of 15 beds (Roberts et al. 2014). In 2011, in-patient admissions in the three hospitals totalled 7993; 32 % of these patients were females while 68 % were males (Roberts et al. 2014). The major diagnoses leading to in-patient admissions included schizophrenia (32 %), substance misuse (26 %), mood disorders (19 %), and neurotic/stress-related disorders (1 %) (Roberts et al. 2014). Disorders such as epilepsy accounted for 6 % of all in-patient admissions. The combined human resource capacity of these hospitals is 1887, including 8 psychiatrists (Roberts et al. 2014). This brings the psychiatrist- to- population ratio to 0.07 per 100,000 persons. There are also 31 non-psychiatrist medical doctors, 21 social workers, 4 occupational therapists, 19 psychologists, 1,256 nurses and 546 other workers (including health assistants and other auxilliary staff) (Roberts et al. 2014). These data suggest that the psychologist- to- population ratio in Ghana is 0.08:100,000 and the psychiatric nurse- to- population ratio is 5.19:100,000 (Roberts et al. 2014). With regards to treatment, only 19 % of patients in the psychiatric hospitals are reported to receive one or more psychosocial interventions, and all the hospitals had at least one psychotropic drug in each therapeutic class (antidepressant, anti-psychotic, mood stabiliser, antiepileptic and anxiolytic drugs) available in their facilities throughout the year (Roberts et al. 2014). However, the hospitals sometimes run out of drugs such as olazipine (an antipsychotic medication), compelling patients to be put on other drugs (Roberts et al. 2014).

The foregoing overview of Ghana’s mental health delivery system shows that there is an urgent need to train more healthcare professionals to support efficient psychiatric care delivery. The low availability of pharmaceutical supplies is also an important concern that needs to be addressed. At the end of the year 2011, the total government spending on mental health delivery was about 1.4 % of the total national health budget (Roberts et al. 2014; World Health Organization 2011). Figures from the Ministry of Health indicate that the mental health sector was allocated a budget of 4,516,163 Ghana cedis (excluding staff salaries) (Roberts et al. 2014). However, a total amont of 5,656,974 Ghana cedis was spent on the delivery of mental health services, showing that the initially-approved funding was less than what was actually needed (Roberts et al. 2014). Most of this amount was spent on operational expenses at the psychiatric hospitals, meaning that costs incurred  in the delivery of psychiatric care in other health facilities were not considered here (Roberts et al. 2014; World Health Organization 2011). Also, no mental disorder is covered by social insurance schemes and there are no social benefits for patients, leaving the financial burden solely to patients, families and carers (World Health Organization 2011). In addition to public funding, some international development partners and non-profit organisations have contributed to mental health delivery in Ghana. The contributions of these agencies have usually been made through the donation of essential drugs (Roberts et al. 2014).

In March 2012, the Parliament of Ghana successfully passed the Mental Health Act into law (Act 846 of 2012) (Doku et al. 2012). The aim was to increase the quality of mental healthcare through improved protection of the rights of patients and clinicians (Doku et al. 2012). However, three years after the passage of this law, there are still challenges and weaknesses in the implementation of policy and legislative frameworks. Among these challenges are (i) insufficient government funding towards the efficient delivery of mental health services (ii) little use of the legal provisions to effectively regulate the detention and handling of patients in health facilities and spiritual healing centres (iii) lack of regulation regarding the practice of psychiatry by traditional and spiritual healers, and (iv) insufficient use of clinical guidelines even where they exist (Doku et al. 2012; Roberts et al. 2014). Moreover, several challenges also exist in terms of mental health service delivery, recordkeeping and records monitoring and evaluation (Roberts et al. 2014; World Health Organization 2011). Mental health information systems are not regularly updated. Similarly, mental health data are not consistently aggregated to provide national reports on the burden of mental health (Roberts et al. 2014).

The mental health policy was formulated and implemented by the Ministry of Health and the Ghana Health Service respectively (World Health Organization 2011). The existing mental health policy was revised in 1996 and refined in 2012 but it still does not address the integration of mental health into primary health care (World Health Organization 2011). However, the policy includes the following components: protection of human rights of patients, human resources, involvement of users and families, advocacy and promotion, equity of access to mental health services across different groups, financing, monitoring system, quality improvement and a list of essential medicines (including anxiolytics, antipsychotics, antidepressants, antiepileptic drugs and mood stabilisers) (Roberts et al. 2014; World Health Organization 2011). The so-called *2007*–*2011 Mental Health Strategy* ensured the revision of the mental health plan and contained a budget, specific goals and timeframes (Roberts et al. 2014). However, due to lack of funds many of the goals have been unattained. Additionally, the country has no emergency or disaster preparedness plan for mental health (Roberts et al. 2014).

### Neuroscience research capacity: challenges and prospects

The foregoing discussion is a clear indication that, while major achievements have been recorded,  research in neuroscience in Ghana needs to improve both in diversity and quantity. Particularly, information is lacking on the (i) epidemiology of many mental and neurological disorders (ii) effectiveness of psychotropic treatments (iii) genetic, genomic and molecular bases of neurological and neuropsychiatric disorders (iv) social, economic and healthcare implications of these diseases, and (v) effectiveness of therapeutic approaches and patient care platforms currently used. In this section, we will highlight some of the challenges that have contributed to the low neuroscience capacity in Ghana. We will also provide some suggestions and discuss current approaches aimed towards building capacity for neuroscience research in Ghana.

### Inadequate expert scientists

Neuroscience is a rapidly evolving field, with implications for social development and economic growth through its applications in areas such as education, business management, sociology, economics, criminal justice, psychology and advertising (Karikari et al. 2015a; Yusuf et al. 2014). Investment in neuroscience education and research is therefore likely to make enormous contributions to building better societies through improved understanding of how the brain works and the application of this knowledge to improve service delivery (Karikari et al. 2015a; Yusuf et al. 2014). However, neuroscience attracts relatively low interests from Ghanaian students and scientists, leading to a disparity in research output between Ghana and other countries (Karikari et al. 2015a, b). This apparent lack of interest is mostly due to challenges such as the lack of research funding, inadequate research infrastructure as well as the lack of degree programmes to prepare students for careers in neuroscience (Karikari et al. 2015a, b). Difficulties in accessing well-resourced neuroscience research facilities in the country may also contribute to the low availability of molecular and genetic studies into neurological and neuropsychiatric diseases. Additionally, the dearth of resident neuroscientists plays a major role in the absence of neuroscience degree programmes in the country, due to the unavailability of experts required to provide world-class student training in this area (Karikari et al. 2015a, b). In order to attract more researchers and students into neuroscience in Ghana, the development of strategies aimed at addressing the infrastructural, training and funding issues will be essential (Karikari 2015a; Karikari et al. 2015a). Ongoing and suggested attempts to address these challenges have been discussed in the sections below.

### Neuroscience research infrastructure and training programmes

Although there are dozens of registered higher education institutions in Ghana, there is no degree programme in neuroscience offered in the country at the moment (Karikari et al. 2015a, b; Karikari and Quansah 2015). This negatively affects the supply of scientists and clinicians with appropriate training in neuroscience to help improve biomedical research and healthcare delivery in the country (Karikari et al. 2015a; Karikari and Aleksic 2015). In order to provide well-resourced facilities for neuroscience research in Ghana, the KBTH in partnership with the Korle Bu Neuroscience Foundation (KBNF; a registered charity dedicated to supporting neurological healthcare, education and research) are working towards the establishment of a proposed Korle Bu Neuroscience Center of Excellence (KBNCE) (Cain 2011). Under KBNCE, a collaborative neuroscience graduate programme has been proposed to provide local opportunities for Ghanaian students to study neuroscience (Cain 2011). This programme, which would be offered at the University of Ghana, is aimed at supporting the future sustainability of the research component of the KBNCE through the development of basic and clinical neuroscience research capacity in the country (Cain 2011). The introduction of interdisciplinary graduate programmes of this nature would greatly benefit the country, by enhancing collaborative research especially those requiring neuroscience expertise (Karikari et al. 2015a, b). Due to the challenging nature of neuroscience education, particularly in resource-limited environments, institutional partnerships between neuroscience and computer science departments would support student training in the use of Internet- and computer-based tools in exploring neuroscience concepts and techniques (Karikari 2015b, c). Aside from degree programmes, practising scientists and clinicians wanting to deepen their neuroscience knowledge in order to advance their research and teaching activities may find the short-term training programmes offered for African scientists useful. Notable examples of these programmes include the following: (i) training programmes in neurogenetics, insect neuroscience, genomics data analysis and open labware provided by the science-based non-profit Teaching and Research in Natural Sciences for Development in Africa (TReND; http://trendinafrica.org) (ii) other neuroscience-focused workshops organised under the sponsorship of the International Brain Research Organization (IBRO) and other organisations to provide practical training in specific neuroscience research areas to African scientists, and (iii) IBRO-funded teaching tools workshops aimed at training faculty members in the best methodology for the effective teaching of neuroscience in Africa (Baden et al. 2015; Juliano 2012; Karikari 2015a; Karikari et al. 2015a; Karikari and Aleksic 2015; Yusuf et al. 2014).

The ability of scientists to undertake high-impact research in neuroscience in Ghana is often hindered by the low availability of appropriate technology, modern equipment and local content in the scientific literature (Awenva et al. 2010; Karikari et al. 2015a). Addressing these challenges would contribute immensely to bridging the existing scientific productivity gap between Ghana and other countries (Awenva et al. 2010). Higher education institutions in Ghana could benefit from training and research equipment donation programmes offered by organisations such as Seeding Labs (http://seedinglabs.org), Adequation (http://adequationgermany.embl.de/), TReND (www.TReNDinAfrica.org) and KBNF (http://kbnf.org/). These organisations support African hospitals, research centres and universities with functional medical and research equipment. Details about how these initiatives work have been provided elsewhere (Karikari et al. 2015a; Yusuf et al. 2014).

Ghanaian scientists could also benefit from travel fellowships and short courses offered or supported by international organisations such as IBRO, the Society for Neuroscience (SfN), the National Academy of Sciences (USA), the United Nations Educational, Scientific, and Cultural Organization (UNESCO), Guarantors of Brain and The World Academy of Sciences to help scientists in developing countries to obtain state-of-the-art neuroscience training. Some of these workshops have been aimed at building scientific capacity in Ghanaian universities, helping to improve teaching and learning of neuroscience. For example, IBRO’s fourth teaching tools workshop, funded by IBRO with support from UNESCO, SfN and NAS was held at the University of Cape Coast Medical School in Cape Coast, Ghana, in September 2011 (Juliano 2012). At this programme, participants selected from all over Africa were trained in how best to teach neuroscience concepts using simple-but-effective approaches (Juliano 2012). In addition, The International Parkinson and Movement Disorder Society and the World Federation of Neurology recently teamed up to organise short training courses for non-neurology specialist physicians in Ghana and elsewhere in West Africa (Cilia 2013). The purpose of this initiative was to help improve the quality of clinical neurology in the sub-continent, by ensuring that more non-specialists are trained to become more competent in diagnosing and treating neurology cases (Cilia 2013). More of such programmes are needed to help improve the capacity of both scientists and clinicians in Ghana in neuroscience.

### Neuroscience research funding

With the existing challenges involved in accessing government funding for research, conducting cutting-edge research to help tackle local health needs is difficult in Ghana (Karikari 2015a). The country lacks established mechanisms to competitively fund research, innovation and technological development (UNCTAD 2011). Notably, the financial contribution of the Government towards basic research is low; for example, only about 0.3 % of the country’s gross domestic product is allocated to basic research (UNCTAD 2011). However, only about 10 % of this fund is used to support actual reseach costs, since about 90 % is spent on staff remuneration and other operational costs (UNCTAD 2011). In addition, there is no government-led competitive research funding programme in Ghana, meaning that scientists often rely on funding from international agencies for their research (Karikari 2015a). However, the research priorities attached to these international funding calls may not be necessarily aligned with the research priorities of Ghana (Karikari 2015a; UNCTAD 2011). Also, the highly-competitive nature of international funding programmes makes it difficult for early-career scientists and senior scientists with low publication records to obtain funding. Concerning funding improvements, the African Union, about a decade ago, challenged all member states to spend 1 % of their gross domestic product on local research and development (Irikefe et al. 2011). However, Ghana has not been able to reach this research funding target (Irikefe et al. 2011; UNCTAD 2011). To ensure that more Ghanaian scientists conduct research that is in line with national research priorities, more funding support from the Government, industries, charity organisations and philanthropists will be necessary (Karikari 2015a; Karikari et al. 2015a; Karikari and Aleksic 2015; Quansah and Karikari 2015; UNCTAD 2011). Increasing financial investment would not only help to ensure the long-term sustenance of neuroscience research in Ghana, but would also help to improve local neurological healthcare. Mechanisms should also be put in place to ensure that governmental funding allocated to higher education institutions are spent specifically on the indicated assignments.

## Conclusion

In this study, we have shown that considerable progress has been made in neuroscience-related research in Ghana over the last two decades. Research interest has centred mainly on hospital and community-based surveys into neurocognitive impairments in non-nervous system disorders, depression and suicide, epilepsy and seizures, neurological impact of substance misuse, and neurological disorders. Importantly, productivity in neuroscience-related research has increased in recent times, with about 60 % of research output over the last two decades recorded in 2009–2015.

Notwithstanding the progress made, more needs to be done to improve future research output in this area. Particular areas that future research should focus on include epidemiology of neurological and neuropsychiatric disorders, effectiveness of treatment options, and the genetic, molecular and genomic basis of these disorders. This might lead to the identification of disease risk factors as well as genetic factors that either protect or predispose individuals to specific diseases, with important implications for clinical intervention and disease management. Research infrastructural constraints, low funding issues, and the lack of expert scientists and neuroscience degree programmes are important issues that need to be addressed to ensure sustainable development of neuroscience in the country.
